# Catastrophic health expenditure and impoverishment in Mongolia

**DOI:** 10.1186/s12939-016-0395-8

**Published:** 2016-07-11

**Authors:** Javkhlanbayar Dorjdagva, Enkhjargal Batbaatar, Mikael Svensson, Bayarsaikhan Dorjsuren, Jussi Kauhanen

**Affiliations:** Department of Health Policy and Management, School of Public Health, Mongolian National University of Medical Sciences, Zorig Street, Ulaanbaatar, 14210 Mongolia; Institute of Public Health and Clinical Nutrition, Faculty of Health Sciences, University of Eastern Finland, Kuopio, Finland; Health Metrics Unit, the Sahlgrenska Academy, University of Gothenburg, Gothenburg, Sweden; Department of Health Systems Governance and Financing, WHO, Geneva, Switzerland

**Keywords:** Catastrophic health expenditure, Impoverishment, Financial protection, Mongolia

## Abstract

**Background:**

The social health insurance coverage is relatively high in Mongolia; however, escalation of out-of-pocket payments for health care, which reached 41 % of the total health expenditure in 2011, is a policy concern. The aim of this study is to analyse the incidence of catastrophic health expenditures and to measure the rate of impoverishment from health care payments under the social health insurance scheme in Mongolia.

**Methods:**

We used the data from the Household Socio-Economic Survey 2012, conducted by the National Statistical Office of Mongolia. Catastrophic health expenditures are defined an excess of out-of-pocket payments for health care at the various thresholds for household total expenditure (capacity to pay). For an estimate of the impoverishment effect, the national and The Wold Bank poverty lines are used.

**Results:**

About 5.5 % of total households suffered from catastrophic health expenditures, when the threshold is 10 % of the total household expenditure. At the threshold of 40 % of capacity to pay, 1.1 % of the total household incurred catastrophic health expenditures. About 20,000 people were forced into poverty due to paying for health care.

**Conclusions:**

Despite the high coverage of social health insurance, a significant proportion of the population incurred catastrophic health expenditures and was forced into poverty due to out-of-pocket payments for health care.

## Background

Ensuring financial protection for the population against the cost of ill-health is one of the fundamental objectives of the health system [1]. It has been estimated that approximately 100 million people worldwide are forced into poverty, and around 150 million people face catastrophic expenditure as a consequence of high out-of-pocket (OOP) payments for health care each year [2]. Additionally, a large number of people abstain from utilizing health care resources due to the financial costs.

The literature has shown that a higher proportion of OOP payments of health expenditure is related to a higher percentage of households that suffer from catastrophic health expenditures and are forced into poverty [3, 4]. The World Health Report 2010 proposed a strategy to improve or modify country-specific health financing systems so as to achieve universal health coverage (UHC), which aims to ensure that everyone has access to comprehensive and health services of acceptable quality without incurring financial hardship [2]. Furthermore, UHC has become one of the overarching health targets of the Sustainable Development Goals by 2030, approved by the UN General Assembly in September 2015 [5].

As in many other countries, health financing reforms in Mongolia are guided by the concept of UHC. Before 1990, Mongolia had a centralised health system where the government was wholly responsible for both health service delivery and financing. It provided access to universal “free” health care (i.e. no patient cost-sharing) [6]. However, during the socio-economic transition from a centrally planned to a market-oriented economy that started in 1990, health financing reforms aimed to expand funding sources beyond the government budget [7]. This policy change encouraged household contribution to health financing through user fee and co-payment initiatives. At the same time, reforming the health care financing system with user fees/co-payments needed a strategy and evidence-based actions where available, so as to preclude people from incurring financial difficulties due to the introduction of co-payments.

In 1994, the government of Mongolia successfully introduced a new social health insurance system (SHI) [8, 9] . The main purpose of SHI introduction and implementation was not only to promote equitable access to health care through prepayment, i.e. reducing negative effects of user fee policies, but also to provide financial protection for the population from excessive financial hardships, especially for low income and vulnerable population groups [7, 9]. This policy focus is still valid, and remains as an issue of priority in Mongolia’s medium- and long-term health and SHI development policies [6, 9–12].

Currently, SHI is one of the main sources of health financing along with the government health budget. As of 2014, almost the entire population is covered by SHI and entitled to the same health service benefits regardless of their socio-economic characteristics [9].

According to the health insurance law, the insurance premium for employees in public and private sectors is 4 % of their monthly salaries, which is shared equally between the employer and employee The premium for children under 18 years and students is equal to 1 % of the national minimum wage per month. For all other groups, the premium rate is 2 % of the national minimum wage. It is worth mentioning that the premiums for some vulnerable and specific groups, including children younger than age 18, pensioners, mothers caring for new-born children up to the age of two, military personnel and people on low incomes are fully subsidized by the government [12].

The benefit package offered by SHI includes major inpatient services at both secondary and tertiary level hospitals with a patient cost-sharing at around 10 to 15 % and a limited number of outpatient services [8, 13]. In addition, SHI covers 50 to 100 % of the cost of essential medicines prescribed by family physicians and medical doctors working at the primary level of health centres [8]. The Government health budget funds primary health care services provided through family health centres and *soum* (district) health centres; and some specific services including treatment of chronic and infectious diseases provided at secondary and tertiary level hospitals [8, 13].

In the past, several efforts have been made to assess the effect of direct out-of-pocket payments on household income and expenditure. The World Bank report, based on the Mongolian Household Socio-Economic Survey (HSES) 2007/2008, stated that the incidence of catastrophic health expenditure was 3.3 %, when the threshold was set at health expenditures at 40 % or more of non-food expenditure. It also reported that the rate of impoverishment due to out-of-pocket payments was 2.5 % [14]. These results indicated that the incidence of catastrophic health expenditure and impoverishment is smaller in Mongolia compared to some other developing countries [14].

Other studies found that the poverty rate in Mongolia has decreased from 35.2 to 27.4 % (between 2008 and 2012) [15, 16]. In the same period, SHI coverage increased from 83.2 to 98.6 % [17, 18]. Despite these positive trends, our previous studies showed that the degree of income-related inequalities in health care utilization increased between 2008 and 2012 [19]. In addition, the poor tended to use primary health care rather than secondary or tertiary hospital care, although they had a lower health status and greater needs [19], i.e. high-income groups reported significantly better health [20]. In contrast, high-income groups were more likely to bypass primary health care and directly choose more costly health services at the higher levels of hospitals or in the private sector [19]. A referral system has been built into the health sector; nonetheless, gatekeeping at primary health care is weak and unnecessary self-referral to the upper level hospitals is common [18].

Another emerging fact is that the share of OOP payments in the total health expenditure reached 41 % in 2011 [9]. As the international evidence indicates, when OOP payments exceed 20 % of total health expenditure, it is difficult to reach UHC, and a country may need to improve financial protection policies [3, 21].

Based on these grounds, this paper aims to contribute to policy discussions by estimating the incidence of catastrophic health expenditure and the rate of impoverishment with the latest available household income and expenditure survey data, and to analyse the overall trends and effects of SHI on financial protection in Mongolia. The paper also intends to promote uniform measurement and regular monitoring of household catastrophic health expenditure and impoverishment in Mongolia as part of national efforts to reform health financing in order to achieve UHC.

## Methods

### Data

Data used in this paper was obtained from the HSES 2012 (http://www.1212.mn/en/), conducted by the National Statistical Office of Mongolia (NSO). The HSES is a nationally representative and annually conducted survey intended to “evaluate and monitor the income and expenditure of households, update the basket and weights for consumer price index, and inputs to the national accounts” [16]. The survey has three levels of strata, including Ulaanbaatar (the capital city), *aimag* (provincial) centres and *soum* (district) centres, and the countryside [16]. The survey used a standard questionnaire which consisted of a wide range of questions on household income, expenditure, consumption as well as health care payments. A total of 12,811 households are included in the survey. The detailed household characteristics were described in the HSES 2012 report [16].

### Measuring catastrophic health expenditure

Catastrophic health expenditure occurs if OOP payments for health care exceed a particular threshold of a household’s resources: income, expenditure or consumption [22, 23].

However, it is well known that there is a limitation in using total household expenditure as an indicator for household resources, considering that low-income households may have low OOP for health care due to the fact that the vast majority of resources are spent on food and basic survival. Thus, if households are not able to meet catastrophic health payments, we may underestimate the burden of OOP payments for health care. A partial solution is to estimate catastrophic health expenditure if health care costs exceeds the chosen threshold of household non-food expenditure [23]. Household non-food expenditures is also known as “non-discretionary expenditure” [22] or “capacity to pay” [24]. It is measured as the difference between total household expenditure and household food expenditure. Hereafter, we use the term “capacity to pay”, and in this paper we use both “total household expenditure” and “capacity to pay” as living standard indicators.

If *x* is the total household expenditure, *nf*(*x*) is capacity to pay, *T* is OOP payments for health care, *z* is a given threshold, and thus a household incurs catastrophic health expenditure if *T*/*x*, or *T*/*nf*(*x*), exceeds z [23].

In the literature commonly used threshold values (z) are 10 % for total household expenditure and 40 % for capacity to pay [23]. However, in this paper we also use additional thresholds in order to investigate potential consequences and robustness in the interpretations of the results.

### Measuring incidence and intensity of catastrophic payments

The incidence (H) of catastrophic payments can be expressed by head count. It is obtained by the proportion of households that incurred catastrophic payments and is estimated by the formula below [23].

1 $$ H=\frac{1}{N}{\displaystyle \sum_{i=1}^N}{E}_i $$

where N is the sample size. *E* is an indicator such that *E*_*i*_ = 1 if $\frac{T_i}{x_i} > z$, and otherwise zero.

The head count cannot capture the magnitude by which household OOP payments exceed the given threshold. While, the catastrophic payments overshoot, *O*, denotes the average extent to which health payments exceeds the chosen threshold for households that incurred catastrophic expenditures. The household overshoot is estimated as follows [23]:

2 $$ {O}_i={E}_i\left(\left(\frac{T_i}{x_i}\right)-z\right) $$

Then, the average of the overshoot is simply written as:

3 $$ O=\frac{1}{N}{\displaystyle \sum_{i=1}^N}{O}_i $$

Briefly, *H* refers to the incidence of catastrophic payments, whereas *O* is the intensity of catastrophic payments. Both measures, *H* and *O*, are insensitive to the distribution of catastrophic payments. It is evident that the consequences of catastrophic health expenditures for rich and poor are different. We used concentration indices, *C*_*E*_ and *C*_*O*_, for *E*_*i*_ and *O*_*i*_, respectively, to measure the distribution of catastrophic payments in relation to household expenditures. The concentration indices fall between -1 and +1. Positive/negative *C*_*E*_ indicates the better-off/worse-off are more likely to exceed the chosen threshold. Analogously, the overshoot is concentrated among the rich/poor if *C*_*O*_ is positive/negative.

The weighted head count and overshoot measures can easily be estimated as follows [22]:

4 $$ {H}^w=H\cdot \left(1-{C}_e\right) $$

5 $$ {O}^w=O\cdot \left(1-{C}_o\right) $$

The weighted head count and overshoot measures show the impact of OOP when different weights are given to households depending on expenditure level [23]. The households with the lowest expenditures are weighted by 2, and the households with the highest expenditures are weighted by 0, and the weight decreases with higher household expenditures.

If the concentration index (*C*_*e*_) is negative, the weighted head count (*H*^*w*^) is greater than the head count (*H*) [23].

All of the measures in terms of capacity to pay were estimated as the replacement of total household expenditure, *x*_*i*_, by capacity to pay, *nf*(*x*_*i*_), in the above indicated equations.

### Health care payments and poverty

A high OOP payment for health care may push households into poverty. In practice, a number of people who are forced into poverty by the need to pay for health services are not included in the national poverty measurement as poor households.

Impoverishment effect of OOP payments for health care can be obtained by the difference between a poverty level with the gross of OOP payments (before health care payments) and a poverty level with the net of OOP payments (after health care payments). First, we estimated the gross of the health payments poverty ratio (*HP*^*gross*^). This gives the percentage of the population living below the poverty line before health payments [23];

6 $$ H{P}^{gross}=\frac{{\displaystyle {\sum}_{i=1}^N}{s}_i{p}_i^{gross}}{{\displaystyle {\sum}_{i=1}^N}{s}_i} $$

where *p*_*i*_^*gross*^ is equal to 1 if the per capita total expenditure of household (*y*_*i*_) is less than the poverty line and otherwise 0. *s*_*i*_ denotes the household size and N indicates the number of households in the sample.

The next measure is a gross of health payments individual-level poverty gap, which is estimated as follows:

7 $$ {g}_i^{gross}={p}_i^{gross}\left( PL-{y}_i\right) $$

PL refers the poverty line. Based on the equation 7, the mean of poverty gap is simply found as:

8 $$ {G}^{gross}=\frac{{\displaystyle {\sum}_{i=1}^N}{s}_i{g}_i^{gross}}{{\displaystyle {\sum}_{i=1}^N}{s}_i} $$

The net of health payments head count can be estimated by replacing *p*_*i*_^*gross*^ with *p*_*i*_^*net*^ in the equation 6. Where *p*_*i*_^*net*^ is equal to 1 if the per capita total expenditure of the household is less than the poverty line and The net of the health payments poverty gap is estimated as the replacement of *g*_*i*_^*gross*^ by *g*_*i*_^*net*^ in the equation 8 with *g*_*i*_^*net*^ = *p*_*i*_^*net*^(*PL* − *y*_*i*_).

A normalized poverty gap, which enables us to make international comparisons across countries with different poverty lines and currency units, is estimated as follows: *NG*^*gross*^ = *G*^*gross*^/*PL*

### Poverty line

In order to estimate the above-mentioned poverty measures, a poverty line should be set. We used the Mongolian national poverty line, which was 118,668 Mongolian tugrik (MNT) per month ($ 201.02 PPP in 2011) in 2012 as defined by the NSO [16]. This poverty line was estimated based on the cost of minimum food requirements per person (2100 calories a day) plus some non-food expenditures.

Second, for international comparisons we also used the $1.90 (PPP in 2011) per day per person poverty line defined by the World Bank for international comparisons. After adjustment of poverty parity exchange, this poverty line is equal to 34,769.6 MNT per month in 2012 price [25].

## Results

### Distribution of OOP

The distributions of health service utilization by expenditure quintiles are shown in Table 1. There were 4062 (29.8 %) households that reported that at least one member of the household was hospitalized during the previous year and 15.6 % of the households used outpatient services at least once in the past month. Results showed that the richer used both outpatient and inpatient health services significantly more than did the poor. The urban households reported more outpatient use (*p* < 0.01) than the rural ones. There is no statistically significant difference (*p* > 0.05) between inpatient use in the urban and rural areas.

**Table 1:** Distribution of health service utilization by expenditure quintile

	Outpatient (*N* = 2097)		Inpatient (*N* = 4062)	
All	15.6 %		29.6 %	
Quintile				
1	11.2 %	*P* < 0.01	23.2 %	*P* < 0.01
2	13.9 %		26.8 %	
3	16.9 %		30.3 %	
4	16.9 %		32.2 %	
5	19.2 %		35.6 %	
Urban	17.0 %	*P* < 0.01	29.8 %	*P* = 0.07
Rural	12.9 %		29.3 %	

**Fig. 1:**
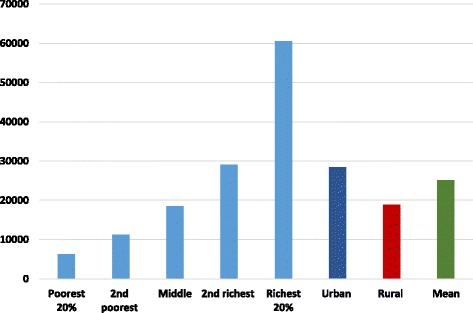
Distribution of OOP health payments (MNT) across quintile

The distribution of OOP health payments (MNT) across quintiles is shown in Fig. 1. The mean OOP payments among the total households was 25,086.5 MNT. The results indicate that the amount of OOP health payments increases with the quintile increase, e.g. the OOP health payments among the richest 20% households were almost 10 times higher compared to the poorest 20% households. In addition, the level of OOP payments in the rural areas was significantly smaller than that in the urban areas.

### Catastrophic health expenditures

The measures of the incidence of catastrophic payments for health care in Mongolia are shown in Table 2. We estimated catastrophic payments as a share of the total household expenditure and capacity to pay, based on several threshold budget shares.

**Table 2:** Percentage of households incurring catastrophic payments for health care in Mongolia, 2012

	Out-of-pocket health spending as a share of total expenditure				As a share of non-food expenditure		
Threshold	5 %	10 %	15 %	25 %	15 %	25 %	40 %
Head count	12.1 %	5.5 %	3.3 %	1.2 %	7.0 %	3.4 %	1.1 %
Concentration index, C_E_	0.03	0.09	0.17	0.32	0.00	0.08	0.29
Rank-weighted head count, H_W_	11.7 %	5.0 %	2.7 %	0.9 %	7.0 %	3.1 %	0.8 %

The incidence of catastrophic head count can be explained as follows. When the threshold is 5 % of the total expenditure, 12.1 % of Mongolian households incurred catastrophic payments for health care. When we increase the threshold, the incidence falls. For instance, 1.2 % of Mongolian households spends in excess of 25 % of the total expenditure on health care. Similarly, as the threshold is raised from 15 to 40 % of capacity to pay, the incidence of catastrophic head count drops from 7 to 1.1 %.

Table 2 provides information on the rank-weighted head counts and concentration index for *Ei*. The results showed that the rank-weighted head counts are smaller than the unweighted head ratio at all levels of thresholds, regardless of whether catastrophic payments is defined by the total expenditure or capacity to pay. It indicates that the better-off are more likely to incur catastrophic payments for health. This was confirmed by positive concentration indices for the incidence of catastrophic payments at all thresholds.

Table 3 shows OOP health payments in excess of catastrophic payments threshold budget share.

**Table 3:** OOP health payments in excess of catastrophic payments threshold budget share in Mongolia, 2012

	Out-of-pocket health spending as a share of total expenditure				As a share of non-food expenditure		
Threshold	5 %	10 %	15 %	25 %	15 %	25 %	40 %
Mean overshoot	0.99 %	0.58 %	0.37 %	0.16 %	0.96 %	0.47 %	0.17 %
Concentration index, C_O_	0.17	0.25	0.33	0.46	0.14	0.24	0.39
Rank-weighted overshoot, O_W_	0.95 %	0.53 %	0.31 %	0.11 %	0.96 %	0.43 %	0.12 %
Mean positive overshoot	8.13 %	10.61 %	11.32 %	12.97 %	13.68 %	14.07 %	15.05 %

Probably the incidence of catastrophic head count, the mean overshoot, falls from 0.99 to 0.16 % as the threshold rises from 5 to 25 %. In the last row of Table 3, the mean positive overshoot (MPO) is provided. It can be read as those spending more than 15 % of the total capacity to pay on health care payments, who on average spent 28.68 % (15 + 13.68 %). Those spending more than 40 % of capacity to pay on health care payments on average spent 55.05 %. On the other hand, the mean overshoot among those exceeding the threshold need is raised as the threshold increases.

### Health care payments and poverty

Poverty measures corresponding to household expenditures both before and after health payments are shown in Table 4. As mentioned before, we used two poverty lines in this study, the Mongolian national poverty line (118,668 MNT) and the World Bank poverty line ($1.90 per day). At the Mongolian national poverty line, the poverty head count is 22.26 %, i.e. 22.26 % of the Mongolian population is estimated to be in poverty using household expenditures as a living standard indicator. After accounting for OOP health care payments, a 0.78 % rise in the poverty head count was observed. This result can be interpreted such that over twenty thousand people in Mongolia are forced into poverty due to OOP health care payments. The poverty gap, the average deficit to reach the poverty line in the population, also rises from 6953.57 MNT to 7284.22 MNT. The normalized poverty gap increased from 5.86 to 6.14 %; however, a slight rise in the normalized mean positive gap was found (26.33 to 26.64 %).

**Table 4:** Measures of poverty based on expenditure gross and net of spending on health care, Mongolia 2012

	The national poverty line 118, 668 MNT per month				The WB poverty line 1.90 US (PPP) per day			
	Gross of health payments	Net of health payments	Absolute	Relative	Gross of health payments	Net of health payments	Absolute	Relative
Poverty head count	22.26 %	23.04 %	0.78 %	3.51 %	0.22 %	0.25 %	0.03 %	15.21 %
Poverty gap (MNT)	6953.57	7284.22	330.65	4.76 %	16.56	20.13	3.57	21.55 %
Normalized poverty gap	5.86 %	6.14 %	0.28 %	4.76 %	0.05 %	0.06 %	0.01 %	21.57 %
Normalized mean positive gap	26.33 %	26.64 %	0.32 %	1.20 %	22.07 %	23.29 %	1.22 %	5.51 %

Using the World Bank poverty line ($1.90 per day), we found that both the poverty head count and poverty gap were much smaller than the results using the Mongolian national poverty line, which is as expected, given that the Mongolian national poverty line is much higher than the World Bank absolute poverty line.

## Conclusion and discussion

Measuring and monitoring OOP impact is critical for countries aiming at UHC. Since there is an increasing OOP concern, this study using the most recent data provides new evidence on catastrophic health expenditure and its impoverishment effect in. In the past, Mongolia made efforts to estimate OOP impacts by using different methodologies and thresholds, which made it difficult to compare and follow-up policy reforms. Some studies estimate the impoverishment effects of health care payments at the individual level using ADePT software, and some do it at the household level [14, 26]. The methodology used in the current study is described in the World Bank guidelines which are more relevant to health financing reforms aimed at improving financial protection [23].

In this study, we estimated the rate of catastrophic health expenditure and impoverishment due to the OOP payments for health care using the HSES 2012. The study reveals several interesting points.

First, 5.5 % of total households suffered from catastrophic health expenditures based on an OOP threshold at 10 % of total household expenditure. At the threshold of 40 % of capacity to pay, 1.1 % of the total household incurred catastrophic health expenditures.

Bredenkamp et al. analysed the incidence of catastrophic health expenditure in Mongolia using the total household consumption as the living standard indicator based on the HSES 2007/2008 data [14]. They found that the incidence of catastrophic health expenditure was 10 and 3.3 % at the threshold of 10 % of the total household expenditure and 40 % of capacity to pay. Considering the different choices of the living standard indicator between the studies, we cannot make a direct comparison between the results. However, it is worth mentioning that the government of Mongolia provided a one-time subsidy to the uninsured groups from the Human Development Fund (a special stabilization fund from mining revenue) in 2011 [6]

Consequently, between 2008 and 2012 the SHI coverage increased from 83.2 to 98.1 %. This implied that specifically among the poor and vulnerable groups, financial protection was extended.

Second, to our knowledge, this study provides the first evidence of intensity of catastrophic health payments in Mongolia. Intensity of catastrophic health payments was relatively low, for instance, it was 0.58 % (0.17 %) at the threshold of 10 % (40 %) of total household expenditure (capacity to pay).

Third, the result demonstrates that the richer households (or households with a higher capacity to pay) are more likely to incur catastrophic health payments. Similar results were reported in the World Bank’s study in Mongolia as well as in other developing countries [4, 14].

This pattern is likely to be a reflection of the health system structure in the country. The ADB (2008) reported that the health system has a risk to become a dual system in where the poor use public facilities and the rich use private facilities [27].

In a previous study, we found that the poor and low-income groups were more likely to use primary health care, regardless of their health needs, which in general are greater than in higher income groups [19, 20]. This may be explained by the fact that primary health care is free of charge and more easily accessible than other upper level hospitals. Today, nearly the entire population has SHI coverage regardless of their socio-economic characteristics; however, the low-income groups are substantially less likely to access specialized health care services at the higher referral levels due to both direct costs, including co-payments, medicines, consultations, and indirect costs, such as for transport and meals.

Primary health care centre have a gatekeeping role in the health sector. In practice, the gatekeeping is weak and cannot control self-referrals to the upper level hospital admissions [6]. In addition, the SHI covers a larger part of inpatient services at the upper level hospitals where the proportion of unnecessary admission is high. These factors lead to higher OOP payments for households.

For instance, in order to obtain inpatient services, the insured person has to pay 10 and 15 % co-payment for inpatient services at secondary and tertiary level hospitals. Moreover, a majority of the inpatients get meals from their home daily. About 40 % of the inpatients buy drugs and injections themselves during their hospitalization [28]. The HSES 2009 reported that 71 % of household OOP payments tend to be made for medications, which were bought from private pharmacies [6, 15].

Fourth, we found that the poverty head count before accounting for OOP payments was 22.26 %, which is lower that the NSO’s estimation (27.4 %) [16]. It can be explained by a choice of living standard indicator. After accounting for OOP for health care from household expenditures, the poverty rate increased by 0.78 percentage points (relative change is 3.51 %). This indicates that about 20,000 people were forced into poverty due to paying for health services based on the Mongolian national poverty line. A similar previous study was conducted by the World Bank team using the HSES 2007/2008, reported that OOP for health payments increased the poverty head count by 2.5 % and that its relative change was 7 % [14]. In general, this impoverishment effect of OOP payments for health expenditures is lower compared to other developing countries, specifically those countries where prepaid health financing mechanisms is less developed [4, 22, 29, 30].

In accordance with the findings of the study, we emphasize some potential policy implications. First, in spite of the developments of financial protection policies and a high SHI coverage, the country still has barriers to reaching UHC. Our results suggest that intensity and incidence of catastrophic health expenditure is relatively low; however, the OOP share of total health expenditures is still 41 %. International and regional evidence showed that the higher share of OOP expenses, in particular above 20 %, of total health expenditures in countries, the more people suffer from catastrophic health expenditure [3, 21]. Additionally, the current way to report the catastrophic health expenditure and impoverishment effect is ad-hoc and is insufficient to support and sustain UHC. Hence, monitoring progress towards UHC in Mongolia requires more frequent studies.

Second, the results also demonstrate that rich households are more likely suffer from catastrophic payments. In general, they tend to bypass primary health care services and seek more expensive upper health care services with self-referral, regardless of their health needs [19]. In this scenario, direct and indirect costs are usually much higher for users owing to the current weak gatekeeping system.

Hence, a more effective referral system may be beneficial, including stronger gatekeeping, at the primary health care level of the health sector. It would not only reduce households’ risk of incurring catastrophic OOP payments for health expenditures, but it would also lead the health sector to better efficiency. For instance, the WHO concluded that primary health care is essential for better health outcomes of those health care systems, in which primary health care plays a main role in i) providing balanced preventive and promotive services regarding the social determinants, and ii) a referral service to higher level of hospitals [31].

Third, it is difficult to control cost escalation and unnecessary treatments in the health sector without improving the regulation of the private health sector, including hospitals and pharmacies. The growth of the private health sector with a weak regulation in the country leads to the unmet health needs among the population and increases duplications of health services in the private and public sectors [9, 27]. Fourth, in 2011, the SHI coverage reached 98.6 % as a result of a one-time subsidy from mining revenues, which was a political promise during the parliament election. Hence, keeping the current high SHI coverage is a vital issue for the future financial protection of the poor and workers in the informal sector.

Importantly, a recent study showed that high SHI coverage alone (breadth) cannot lead to UHC and improve financial protection in the country [9]. There is a need of policy actions to improve other dimensions of UHC, including health service coverage (depth) and cost coverage (height).

Additionally, it is known that people sell their apartments or borrow money from others in order to afford some specific and expensive health care services regardless of the SHI coverage in the country. In this case, intensity of catastrophic health expenditure and impoverishment is much deeper among certain specific groups. Thus, we emphasize that the HSES questionnaire should be extended with questions which focus on how people make health care payments, for example, from either savings or by loan, etc. Further studies aiming to acquire this information will provide more specific policy messages to the decision makers.

Finally, we should note the limitations of this study. The study did not address the distribution or structure of catastrophic health expenditures and its poverty effect. It may call for further studies in this field. In addition, it is a cross-sectional study, thus unable to make a causal analysis.

## Abbreviations

ADB, Asian Development Bank; HSES, Mongolian Household Socio-Economic Survey; MNT, Mongolian tugrik; NSO, National Statistical Office of Mongolia; OOP, out-of-pocket payment; SHI, social health insurance; UHC, Universal Health Coverage; UN, United Nations; WHO, World Health Organization
